# The prognostic role of pretreatment epidermal growth factor receptor T790M mutation in advanced non-small cell lung cancer patients treated with EGFR tyrosine kinase inhibitors

**DOI:** 10.18632/oncotarget.16222

**Published:** 2017-03-15

**Authors:** Guangzhi Ma, Jing Zhang, Liyuan Yin, Hai Jiang, Weiwei Zhang, Yanlin Song, Ming Liu

**Affiliations:** ^1^ Department of Medical Oncology, West China Hospital, Sichuan University, Chengdu 610041, P. R. China; ^2^ Department of Thoracic Surgery, West China Hospital, Sichuan University, Chengdu 610041, P. R. China; ^3^ Department of Neurosurgery, West China Hospital, Sichuan University, Chengdu 610041, P. R. China; ^4^ Department of Orthopedic Surgery, West China Hospital, Sichuan University, Chengdu 610041, P. R. China; ^5^ Department of Endocrinology and Metabolism, West China Hospital, Sichuan University, Chengdu 610041, P. R. China

**Keywords:** pretreatment T790M, NSCLC, EGFR TKI, prognosis, meta-analysis

## Abstract

**Purpose:**

The outcome of pretreatment epidermal growth factor receptor (EGFR) T790M mutation in EGFR mutant non-small cell lung cancer (NSCLC) patients treated with EGFR tyrosine kinase inhibitors (TKIs) is controversial, this study aimed to evaluate the prognostic role of pretreatment T790M in advanced NSCLC patients treated with EGFR TKIs.

**Results:**

A total of 7 eligible studies containing 179 cases and 281 controls were included in the meta-analysis. The pooled hazard ratios (HRs) for progression-free survival (PFS) and overall survival (OS) were 2.21 (95% CI 1.49-3.29, P<0.001) and 1.24 (95% CI 0.90-1.71, P=0.186), respectively. We also did subgroup analyses on OS and PFS according to patients from various districts.

**Methods:**

Identified literatures from various databases were reviewed. A meta-analysis was performed to evaluate the prognostic role of pretreatment EGFR T790M in advanced EGFR mutant patients treated with EGFR TKIs.

**Conclusions:**

Pretreatment T790M may be a poor prognostic factor for PFS in advanced NSCLC patients treated with EGFR TKIs. However, no significant prognostic effect was found between pretreatment T790M mutation and OS. More studies are needed to demonstrate the prognostic role of pretreatment T790M mutation in advanced NSCLC patients.

## INTRODUCTION

Lung cancer is the leading cause of death worldwide, causing 1.6 million deaths with over 1.8 million new cases reported annually [1]. Its outcome remains unsatisfactory with a 5-year survival rate of 13%-16% in Europe and USA [2]. Among lung cancer patients, approximately 85% were non-small cell lung cancer (NSCLC) [3]. Combined chemotherapy, adjuvant radio therapy and first-line target-therapy are the major alternatives for patients with advanced NSCLC [4].

Epidermal growth factor receptor tyrosine kinase inhibitors (EGFR TKIs), including erlotinib and gefitinib, have been proven clinically effective in the treatment of advanced NSCLC harboring EGFR mutations (deletion in exon 19 or L858R) [5]. Several clinical trials found that advanced NSCLC patients harboring EGFR mutations tended to have an improved progression-free survival (PFS) to TKIs compared with chemotherapy as first-line treatment [6, 7]. However drug resistance eventually occurred to the vast majority individuals who underwent continuous TKIs treatment within 10 months on average [8]. Mechanisms of drug resistance was complicated and unclear in some cases. Genetic alterations such as amplification of c-met and over-expression of hepatocyte growth factor (HGF) resulted in 20% of drug-resistant cases [9], and T790M mutation in exon 20 of EGFR gene accounted for 50%-60% patients who developed drug resistance to TKIs [10, 11]. T790M was first reported in 2005 and later proved to be one mechanism causing TKIs drug resistance [10, 12]. But the reason for the high presence of T790M mutation in TKIs post-treatment patients remains unclear. One mechanism suggested that de novo T790M exists in a very minor part of pretreatment tumor cells, which remain vial after TKIs treatment and proliferate rapidly until TKIs no longer validate [13, 14]. Methods such as CH (colony hybridization) assay are highly sensitive to detect T790M mutation out of pre-TKIs patients [15]. Another theory indicated T790M was more an acquired secondary mutation during TKIs treatment, due to the positive-rate rise between the pre-treatment and post-treatment samples [16]. Accordingly, advanced NSCLC patients treated with TKIs harboring T790M mutation should lead to worse prognosis, first reported by Maheswaran et al. [17] in 2008. Among the reported 26 TKIs-naïve patients, 10 patients with detectable T790M showed a shorter PFS (median 7.7 months) compared to the 16 patients without T90M mutation (median 16.5 months). Similar correlation results were reported by some other researchers [13, 18]. However, in a study of 38 patients, with high sensitive CH assay, Fujita et al. found that the presence of T790M mutation indicated favorable outcome in advanced NSCLC patients treated with TKIs [15]. Thus, the prognostic value of pretreatment T790M mutation in advanced NSCLC patients treated with TKIs remains inconclusive.

Due to the controversy, we aimed to perform a meta-analysis to investigate the prognostic role of pretreatment T790M mutation in advanced NSCLC patients treated with TKIs.

## RESULTS

### Study selection

**Figure 1:**
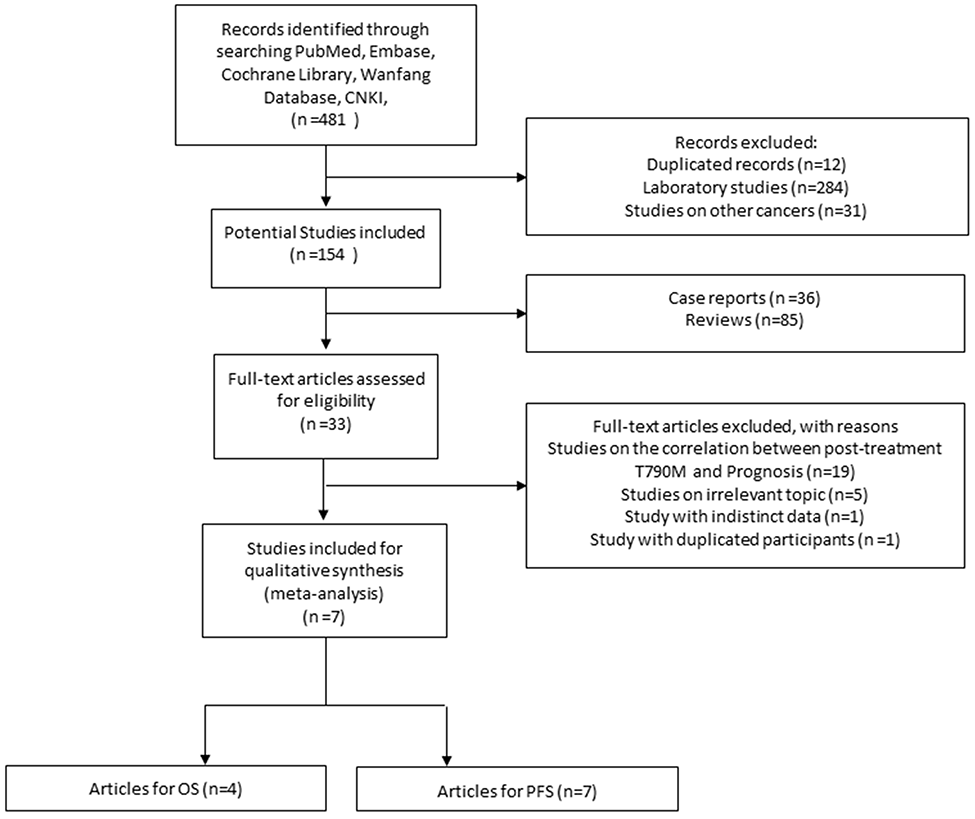
Selection process of studies

In total, 481 relevant citations were retrieved from initial search. After screening the title and abstract of each identified literature, 474 studies were excluded for the reasons as follows: duplicate studies (n=12), studies on other cancers (n=31), laboratory studies (n=284), case reports (n=36), review articles (n=85). Full text of 33 potential studies were reviewed and 26 of them were further excluded: 19 focused on the correlation between posttreatment T790M and prognosis of TKIs-treated patients, 5 studies evaluated irrelevant topics such as response rate, 1 study was excluded due to duplicated trial participants, 1 study was excluded for lacking precise data. Eventually 7 publications [13, 15, 17–21] matched our inclusion criteria. The selection process was shown in Figure 1.

### Study characteristics

Four studies were from East Asia and the rest three studies were from the USA and Spain. A total of 460 participants were included and all harbored EGFR mutations and were either treated with gefitinib or erlotinib. 179 of them had T790M mutation before receiving EGFR TKIs. Tumor specimens were from biopsies in 5 trials or surgery-resected tissues in 2 trials. Every patient was confirmed with advanced NSCLC and histology results indicated 3 studies all dealt with adenocarcinomas while the other 4 studies contained a few cases of other types of lung cancer with the majority of adenocarcinomas. Detection methods for T790M varied in different trials with different positivity rate: allele-specific selective androgen-receptor modulators (SARMs) assay (38.5%) [17], TaqMan Assay (41.8%) [18, 21], Colony Hybridization (CH) Assay (78.9%) [15], MALDI-TOF MS (41.1%) [13], Mass ARRAY System (MS) Assay (25.0%) [20] and ACB-ARMS PCR (22.2%) [19]. Relevant smoking history and median age both were provided. The basic characteristics of the included studies were shown in Table 1.

**Table 1:** Main characteristics of the included studies

First author	Year	Country	Study design	N (F/M)	Number of T790M mutation	Mean age	Clinical stage	Detection method	Specimen	Histology	Treated Drugs	Smoking History
Maheswaran	2008	USA	retrospective	26—	10	—	advanced	SARMS	tumor-biopsy	ADC	Erl. /Gef.	—
Rosell	2011	Spain	retrospective	129(93/36)	45	median 67	advanced	TaqMan®	tumor-biopsy	ADC	Erl.	—
Fujita	2012	Japan	retrospective	38(23/15)	30	median 63	advanced	CH	surgery	ADC	Gef.	—
Su	2012	China (Taiwan)	retrospective	56—	23	—	IIIB or IV	MALDI-TOF MS	tumor-biopsy	ADC/ Other	Erl. /Gef.	—
Costa	2014	Spain	retrospective	60(34/16)	34	—	IIIB or IV	TaqMan®	tumor-biopsy	ADC/SCC/ Other	Erl.	—
Lee	2014	Korea	retrospective	124(74/50)	31	—	IIIB or IV	MS	surgery	ADC(120), NADC (4)	Erl. /Gef.	82never/ 42ever
Zhao	2016	China	retrospective	27(15/12)	6	—	IA-IIIA: 5, IIIB-IV: 22	ACB-ARMS	tumor-biopsy	ADC(26), SCC(1)	Gef.	20never/ 7ever

N: Number of patients; F: Female; M: Male; SARMS: Scorpion Amplification Refractory Mutation System; TaqMan®: TaqMan assay; CH: Colony Hybridization; MS: Mass Spectrometry; ACB: Allele-Specific Competitive Blocker; ADC: adenocarcinoma; NADC: Non-adenocarcinoma; SCC: Squamous cell carcinoma; Erl: Erlotinib; Gef: Gefitinib.

### Meta-analysis results

**Figure 2:**
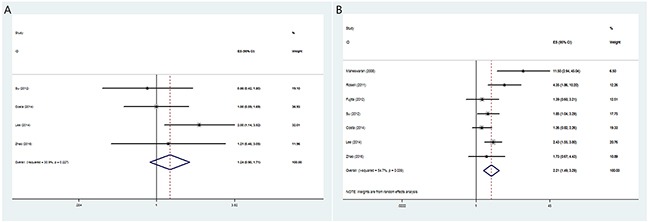
The pooled hazard ratio (HR) for overall survival **(A)** and progression-free survival **(B)** in pretreatment EGFR T790M advanced NSCLC patients treated with EGFR TKIs.

All the seven studies examined the association of T790M mutation and PFS. The HR for PFS was 2.21 (95% CI 1.49-3.29, P<0.001), indicating T790M mutation was associated with worse PFS (Figure 2). The heterogeneity was significant (I^2^=54.7%, P=0.039) and random-effects model was used to pool data.

Four studies [13, 19–21] examined the OS and the pooled HR was 1.24 (95% CI 0.90-1.71, P=0.186) (Figure 2). The heterogeneity was not significant (I^2^= 30.9%, P= 0.227) and fixed-effects model was used.

### Subgroup analysis

Then we performed subgroup analysis on varied districts (Asian/None-Asian). Pre-existed T790M mutation was found to have a negative impact on PFS in patients treated with EGFR TKIs in both Asian (HR=2.01, 95% CI 1.48-2.74, I^2^=0%, P<0.001) and Non-Asian group (HR=3.60, 95% CI 1.10-11.82, I^2^=82.7%, P=0.035). However no statistically significant association between pretreatment T790M mutation in patients treated with EGFR-TKIs and OS in Asian was found (HR=1.41, 95% CI 0.94-2.11, I^2^=39.4%, P=0.095).

The pooled results were shown in Table 2.

**Table 2:** Meta-analyses of pretreatment EGFR T790M to predict the survival outcome of advanced EGFR-mutant NSCLC patients treated with EGFR TKIs

	N of studies	Model	HR (95% CI)	Log-rank p	Heterogeneity (p,I^2^)	Publication Bias	Conclusion
total-PFS	7	Random	2.21(1.49-3.29)	<0.001	0.039, 54.7%	0.230	Positive
total-OS	4	fixed	1.24(0.90-1.71)	0.186	0.227, 30.9%	1.000	Negative
Asia-PFS	4	fixed	2.01(1.48-2.74)	<0.001	0.658,0.0%	0.308	Positive
Asia-OS	3	fixed	1.41(0.94-2.11)	0.095	0.192,39.4%	1.000	Negative
non-Asia-PFS	3	Random	3.60(1.10-11.82)	0.035	0.003,82.7%	0.296	Positive
non-Asia-OS	1	—	—	—	—	—	—

PFS: progression-free survival; OS: overall survival; N: number; HR: hazard ratio; CI: confidence interval.

### Publication bias

**Figure 3:**
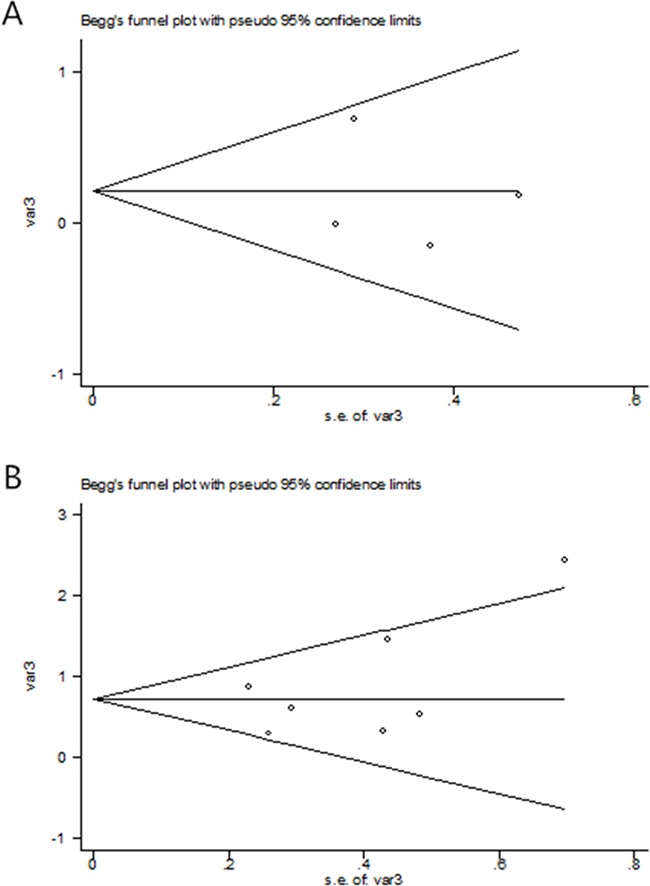
The Begg's publication bias plot of the studies that reported the correlation between OS **(A)** and PFS **(B)** de novo T790M in advanced NSCLC patients prior to EGFR TKIs.

As shown in Table 2 and the plots of publication bias in Figure 3, no publication bias (P=0.23) was found in this meta-analysis.

## DISCUSSION

The present study was designed to evaluate the prognostic role of pretreatment T790M mutation in advanced NSCLC patients treated with TKIs (erlotinib or gefitinib). In this meta-analysis of 7 trials including 179 cases and 281 controls, we found the correlation negative between presence of T790M mutation prior to EGFR TKIs treatment and PFS. Accordingly in our conducted subgroups, whether Asian or non-Asian patients with pretreatment EGFR T790M mutation tended to have a poorer PFS when treated with EGFR TKIs. However, no significant impact was indicated on pooled OS when EGFR T790M mutation existed before EGFR TKIs treatment, and the summarized OS in Asian patients was also not statistically significant.

Despite the pooled results, there are few issues that need to be discussed. Compared with other pooled studies, the study by Fujita et al. [15] was the first and only study which indicated a positive correlation between pretreatment T790M patients and prognosis on PFS in advanced NSCLC patients treated with EGFR TKIs. This study analyzed a different stage when the specimens were obtained (during curative surgeries) and this could, at least in part, explain the difference between this study and the others. Interestingly, several studies have also proven favorable outcomes in TKIs resistant patients when T790M mutation coexisted [22, 23]. Besides, although pretreatment EGFR T790M mutation had an adverse impact on PFS among EGFR TKIs treated patients, no correspondent result was drawn on OS. To account for results that PFS was improved, indolent growth of T790M mutant cells might be involved [24]. Possible explanation might be that although T790M mutation changed the partial growth of some cells, but didn't affect patients’ status as a whole, and importantly, organ metastasis that caused most cancer death wasn't evitable. Such explanation was suggestive, yet corresponding evidence is still deficient. Lack of data could also led to the insignificant result on pooled OS.

One similar meta-analysis was conducted by Ding et al. [25] previously. Their work included 4 trials, which were included in our meta-analysis as well. They found that advanced NSCLC patients with pre-existed EGFR T790M mutation had a poorer PFS, and such results match our study. We included three more studies and examined the pooled OS as well. Furthermore, we have enough studies to do subgroup analysis and analyzed both Asian and non-Asian group.

EGFR T790M mutation was first reported by Kobayashi et al. [10]. They found a threonine-to-methionine amino acid change at the position 790 of EGFR gene. Such change caused structure alternation of tyrosine kinase domain, eventually resulted in affinity declination of hydrogen bonding between EGFR TKIs and its kinase domain. This finding proved as one possible mechanism causing EGFR TKIs drug resistance. One question that needs to be asked is how T790M emerged in NSCLC patients.

T790M was considered a rare EGFR mutation in pretreatment EGFR TKIs patients with advanced NSCLC [26]. De novo T790M was detected in a small proportion of TKIs-naïve patients (0-2%) compared to samples which eventually harbored drug resistance (50%-60%) [27–29]. Therefore most researchers tended to believe T790M was an acquired mutation [8, 10, 29]. However, such explanation tends to overlook the fact that de novo T790M mutation was found in 38.9% of TKIs-naïve cases according to our study. T790M existed in a minor proportion of tumor cells could have amplified rapidly as a dominant allele during TKIs treatment [16]. Our result supports such theory, which could also explain the high-frequent emergence of T790M mutation (50%-60%) after TKIs drug resistance [10, 11]. Yet few studies have been conducted to discuss the topic. The mechanism of T790M emergence could be more complicated than our expectation, which still need to be further explored.

To discuss the prognostic role of pretreatment T790M in advanced NSCLC patients, a reliable detection method is also indispensable when conducting relevant study. Maheswaran et al. found T790M pre-existed in 38.5% of their samples using SARMS [17]. Their finding suggested that the emergence of de novo T790M could be much more frequent compared to previous studies. On the other hand, interestingly, Fujita et al. [15] too initially used SARMS without detecting any positive cases, while CH assay had a positivity rate of 78.9% in total. Therefore, whether de novo T790M existed among TKIs pretreatment patients could be misjudged due to sensitivity of different detection methods.

There are several limitations in our meta-analysis. First of all, the heterogeneity was significant between our 7 studies on survival time and the presence of T790M. Clinical stage were incoherent in each studies and the use of TKIs as first line or after multiple line therapy during the course of cancer treatment could also result in heterogeneity. Varied detection methods could have caused such consequences. Although CH assay [15] had the highest positivity rate among the studies yet to our knowledge we can't decide which assay is most reliable. Secondly, limited studies were conducted currently on the topic between EGFR T790M mutation and prognosis of advanced EGFR-mutant NSCLC patient treated with TKIs. Therefore we should be cautious when referring to the pooled result. Despite of different methods for detection, due to lack of background information, we failed to perform subgroup-analysis such as gender, race, smoking history, drug dosage and individuals of each TKIs (gefitinib or erlotinib), which might contribute to the result as well. However, through detailed protocol, random-effect model and carefully pooled statistics, bias was managed to constrain to the minimum, and the result of the study is guaranteed reliable. Furthermore, although in this study no publication bias was found, publication bias was a major concern for all meta-analyses and could not be completely excluded. It's challenging to deal with patients who went progression after EGFR TKIs resistance caused by T790M and few therapies were effective. Our finding suggested the sensitive detection for T790M in the minor part of tumor cells could be predictive for patient's outcome and various strategies should be reconsidered when dealing with such cases.

To conclude, pretreatment EGFR T790M mutation could have a negative impact on PFS in advanced NSCLC EGFR-mutant patients treated with TKIs (erlotinib or gefitinib). No correlation between the presence of T790M mutation in patients with EGFR mutation prior to EGFR TKIs and OS is found in our study. Our finding suggested the sensitive detection for T790M in the minor part of tumor cells could be indicative for patient's outcome. Therapeutic options should be reconsidered when dealing with such cases, and to date Osimertinib (AZD9291) as a potent irreversible EGFR TKI is the most reliable choice in advanced NSCLC patients with T790M mutation [30–32]. Further studies are needed conducted with high sensitive detection methods for mutation assay to discuss the correlation between T790M and clinical outcome of advanced NSCLC patients treated with TKIs, and the prognostic role of T790M could be re-valued when abundant clinical evidence emerged by then.

## MATERIALS AND METHODS

### Literature search

Two reviewers (GM and JZ) independently searched PubMed, Embase, Cochrane Library, Wanfang Database and China National Knowledge Infrastructure (CNKI) up till October 10^th^, 2016 for eligible studies. The search terms introduced are as followed: “Pretreatment” and “Epidermal Growth Factor Receptor” and “TKIs” and “T790M” and “NSCLC” or “Non-Small Cell Lung” or “Pulmonary Carcinoma” or “Lung Cancer” and “Survival” or “Outcome”.

### Inclusion criteria

1. The studies included patients with advanced NSCLC; 2. The studies examined the presence of pretreatment T790M mutation; 3. The studies investigated the association between the pretreatment T790M mutation and patient survival; 4. NSCLC patients were treated with EGFR-TKIs; 5. Enough survival data were reported to calculate the log hazard ratio (logHR) and variance with methods developed by Parmar, Williamson and Tierney [33–35].

### Exclusion criteria

1. Case reports; 2. Reviews or abstracts; 3. Studies on animals or *in vitro* studies; 4. Studies without enough data.

### Data extraction

Extracted basic information was as follows: name of first author, publication year, country, median age, number of cases involved in the trials, gender, smoking history, clinical stage, T790M detection method, treated drugs, specimen and histology. The data for calculation included hazard ratio (HR) with 95% confidential interval (CI) for PFS and overall survival (OS) or the survival curves with P values.

The literature selection and data extraction process were performed independently by two reviewers (GM and JZ), with any discrepancies being discussed.

### Statistical analysis

Either the HR with 95% CI or the survival curves with P values was used to calculate the logHR and variance for aggregation. Adjusted HR was used if adjusted and unadjusted HRs both existed. Subgroups are divided due to different regions (Asian, Non-Asian). Heterogeneity assumption of HRs was calculated by chi-square based Q-test and I^2^ statistic test [36]. The fixed-effect model (the Mantel-Haenszel method) [37] was applied for HR calculation, if the heterogeneity among studies was not considered statistically significant (I^2^<50% or P>0.10). Otherwise the pooled HR should be evaluated by the random-effect model. Additionally, the potential publication bias was assessed by funnel plots using the methods described by Begg's et al. [38]. If the P value was no more than 0.05 then it's considered statistically significant in publication bias [39]. The STATA (version 11, Stata Corporation) was used to perform our data analysis.
